# A Review of Very-Low-Birth-Weight Infants Admitted to the Kangaroo Mother Care Unit in Johannesburg, South Africa

**DOI:** 10.7759/cureus.20428

**Published:** 2021-12-15

**Authors:** Tanusha Ramdin, Michael Radomsky, Christina Raxendis, Tejis Devchand, Cassady Morris, Charmaine Sekgota, Lorenzo Stols, Mantoa Mokhachane

**Affiliations:** 1 Paediatrics and Child Health, University of the Witwatersrand, Johannesburg, ZAF; 2 Unit for Undergraduate Medical Education, University of the Witwatersrand, Johannesburg, ZAF

**Keywords:** infection, mortality, weight gain, limited resources, very low birth weight

## Abstract

Background

Kangaroo Mother Care (KMC) is a widely implemented intervention developed as an alternative form of care in low- and middle-income countries (LMICs) for neonates. The implementation of KMC has significantly reduced morbidity and mortality in very-low-birth-weight infants (VLBWIs).

Aim

To describe the maternal and neonatal characteristics and clinical outcomes in VLBWIs who received KMC at a tertiary hospital.

Methods

This is a retrospective descriptive study of 981 VLBWIs admitted at Charlotte Maxeke Johannesburg Academic Hospital (CMJAH) over a six-year period (January 1, 2014, to December 31, 2019).

Results

The mean gestational age of infants admitted to the unit was 29.6 weeks (standard deviation (SD): 2.4), with a mean birth weight of 1185 g (SD: 205.6). The average duration of admission in the neonatal unit was 37 days. The mean rate of weight gain was 37.6 g/kg/day (SD: 57.6). The majority of infants were breastfed (61.4%). In our study, the prevalences of the complications of prematurity were as follows: respiratory distress syndrome (RDS), 84.2%; late-onset sepsis (LOS), 26.1%; and retinopathy of prematurity (ROP), 10.6%. The mortality rate was 3.1%. Maternal comorbidities include human immunodeficiency virus (HIV) (26.4%), syphilis (2.9%) and gestational hypertension (33.7%). The antenatal clinic attendance rate was good (84.7%).

Conclusion

KMC is a cost-effective alternative to conventional care for VLBWIs in limited-resource countries, with evidence of increased weight gain, less rates of complications of prematurity and low overall mortality. The provision of KMC facilities is urgently required in LMICs.

## Introduction

Premature birth before 37 weeks of gestation and the associated low birth weight are direct causes of neonatal morbidity and mortality worldwide, as well as major risk factors for other causes of mortality in this group, e.g., sepsis [1]. The rates of low birth weight and prematurity have been estimated to be between 10% and 20% globally, accounting for approximately 20 million births per year [2,3]. This high prevalence has led to the implementation of alternative care strategies, including Kangaroo Mother Care (KMC).

KMC was first described formally in 1978 by Dr. Edgar Sanabria, a neonatologist in Colombia, as an alternative to incubators in low-resource settings [4]. It has since been widely adopted in a range of settings, including those with little resource restriction, as the benefits extend beyond the initially described homeostasis of the infant [5].

The three key components of KMC are the kangaroo position (continuous skin-to-skin contact between the mother and the infant), exclusive breastfeeding (when possible), and early discharge and careful follow-up [6].

KMC is thought to be beneficial up to the point that the infant can no longer tolerate it, which is generally around 37 weeks of gestational age [6].

The nutritional aspect of KMC aims to achieve a rate of weight gain similar to that of a third trimester, intrauterine foetus, which is approximately 15-20 g/kg/day [6]. Two-hourly exclusive breastfeeding is preferred, but not necessarily attainable in all settings. A special emphasis on maternal support and coaching is made to ensure optimal technique [6].

The early discharge of infants who qualify for KMC is not used universally. The initial suggestion was discharge as soon as the kangaroo position and feeding had been perfected by the mothers [7]. Follow-up would then be daily until full-term corrected age had been reached [6,7]. The follow-up is likely more consistent in resource-rich countries and thus is not necessarily a part of the KMC protocol of some facilities.

Overall, previous comparisons of KMC with conventional care have shown significant, cause-specific decreases in morbidity and mortality of neonates who receive KMC [1-5].

Improvements in temperature regulation are the core feature, with a reported increase of neonate body temperature of 1°C in a two-hour session [6]. Another vital system homeostasis has been described previously, including the stabilisation of heart and respiratory rates and oxygen saturation levels and decrease of apnoeic episodes [5,6,8].

A decreased rate of sepsis has also been described, with one study reporting a 47% lower risk [5]. One postulated reason for this may be the transfer of maternal commensal organisms to the infant during close contact [5].

The psychological benefits of KMC have led to its implementation in resource-rich settings. The early, continuous skin-to-skin contact enhances the parent-infant relationship [9]. Additionally, mothers reported feeling more competent and empowered, with decreased rates of mothering disability syndrome (feelings of worry, stress and incompetence) [9].

The long-term outcomes of patients who received KMC in infancy have been studied in recent years. It has been noted that growth parameters and neurological sequelae, including IQ, sensorial or motor morbidity, were not significantly different from conventional care infants [2,10]. An interesting outcome described was that ex-KMC patients had less social anxiety and aggressive behaviour and were less impulsive and hyperactive [2]. A potential reason postulated for this was that the mothers were better trained and more sensitive to the needs of their children.

The benefits of Kangaroo Mother Care are varied and well documented, making it an appropriate alternative tool in the care of neonates across all social settings.

## Materials and methods

Aim

This study aimed to review the clinical characteristics and outcomes of very-low-birth-weight infants (VLBWIs) who receive KMC at a tertiary hospital in Johannesburg, South Africa.

Study design

This study was a retrospective descriptive study conducted in the KMC unit of a tertiary hospital. The unit has 10 beds and requires mothers to stay with their neonates continuously, with six-hourly nursing observations done.

Inclusion criteria

All VLBWIs admitted to the KMC unit between January 1, 2014, and December 31, 2019 (six-year period), were included in the study.

The specific admission criteria for the unit include haemoglobin greater than or equal to 8 g/dL, current weight of 1200 g or more with evidence of weight gain, tolerance of full feeds and clinical stability on room air (i.e., no supplemental oxygen requirements).

Exclusion criteria

Patients with missing or incomplete data were excluded.

Statistical methods

Data were collected from an existing database using Research Electronic Data Capture (REDCap). It was captured and analysed using Microsoft Excel 2016.

Both maternal and neonatal factors were analysed. Neonatal variables included birth weight, gestational age, APGAR score, sex, duration of admission, feeds (breastfed, formula-fed or mixed feeds), rate of weight gain and presence of any complications of prematurity. The complications of prematurity included were respiratory distress syndrome (RDS) (and requirement of adjuvant therapies including nasal continuous positive airway pressure (NCPAP) and surfactant), necrotising enterocolitis (NEC), retinopathy of prematurity (ROP), patent ductus arteriosus (PDA) and late-onset sepsis (LOS).

Maternal variables included mode of delivery, attendance of antenatal care, use of antenatal steroids and maternal comorbidities (human immunodeficiency virus (HIV), syphilis, hypertension and chorioamnionitis).

Definitions and criteria

Hypertension: A diastolic blood pressure ≥ 90 mmHg but <110 mmHg on two occasions taken at least two hours apart or a single diastolic measurement of ≥110 mmHg, and/or a systolic blood pressure ≥ 140 mmHg but <160 mmHg on two occasions taken at least two hours apart or a single systolic measurement of ≥160 mmHg. A raised systolic pressure is indicative of hypertension, even in the absence of a raised diastolic blood pressure [11].

Low birth weight: Any birth weight less than 2.5 kg.

Very-low-birth-weight infants (VLBWIs): between 1 and 1.5 kg. 

Extremely low-birth-weight infants (ELBWIs): less than 1 kg.

Maternal chorioamnionitis: Classified as maternal temperature above 37.8°C and any two of the following: foetal tachycardia, offensive liquor or maternal leukocytes of >12,500/mm^3^ (Gibbs criteria) [12].

Maternal (gestational) hypertension: New onset of hypertension presenting only after 20 weeks of gestation without significant proteinuria.

Necrotising enterocolitis: Staged using the modified Bells’ criteria. NEC grade 2 or 3 were included [13].

Premature: Any infant born before or less than 37 weeks of gestation.

Late-onset sepsis: An infection of the bloodstream or meninges proven by culture at least three days (72 hours) after birth.

## Results

Over the six-year period defining this study, a total of 981 infants were admitted who met the criteria for analysis. Baseline maternal and neonatal demographic characteristics are shown in Table 1.

Neonatal characteristics

The mean gestational age for infants admitted to the unit was 29.6 weeks (standard deviation (SD): 2.4), with a mean birth weight of 1185 g (SD: 205.6). The majority were female (57.3%). The average duration of admission was 37 days, with a wide range from 0 to 169. The calculated average rate of weight gain was 37.6 g/kg/day (SD: 57.6). The median five-minute APGAR score was 8.

The rates of various complications of prematurity are listed below in Table 2. The rates of RDS are 84.2%, with the majority of patients requiring NCPAP (67.3%) and at least one dose of surfactant during admission (63.8%). Of those screened for intraventricular haemorrhage (IVH) and ROP, 25.5% had grade 1, 2, 3 or 4 IVH and 10.6% had any stage of ROP.

**Table 1:** Baseline characteristics of neonatal and maternal demographics of VLBWIs admitted to KMC at a tertiary hospital in Johannesburg, South Africa VLBWIs: very-low-birth-weight infants; KMC: Kangaroo Mother Care; SD: standard deviation; HIV: human immunodeficiency virus

Neonatal Characteristics	
Birth weight in grams, mean (SD)	1185.17 (205.6)
Gestational age in weeks, mean (SD)	29.6 (2.4)
Weight gain in gram/day, mean (SD)	37.6 (57.6)
Maternal Characteristics	
Total: n = 981	Frequency (%)
Delivery by caesarean section	665 (67.8)
HIV infection	259 (26.4)
Syphilis infection (n = 971)	28 (2.9)
Gestation hypertension	331 (33.7)
Chorioamnionitis	32 (3.3)
Antenatal care	831 (84.7)
Antenatal steroids	515 (52.5)

**Table 2:** Complications of prematurity of VLBWIs admitted to KMC at a tertiary hospital in Johannesburg, South Africa VLBWIs: very-low-birth-weight infants; KMC: Kangaroo Mother Care; NCPAP: nasal continuous positive airway pressure

Complications of Prematurity	
Total: n = 981	Frequency (%)
Respiratory distress syndrome (n = 976)	822 (84.2)
NCPAP (n = 912)	614 (67.3)
Surfactant administration on admission (n = 972)	620 (63.8)
Late-onset sepsis (n = 978)	255 (26.1)
Patent ductus arteriosus (n = 976)	71 (7.3)
Blood transfusion (n = 967)	332 (34.3)
Abnormal cranial ultrasound findings (n = 981)	603 (25.5)
Feeds on discharge (n = 944)	
Breastfeeding	576 (61.4)
Formula feeds	318 (33.7)
Mixed feeds	46 (4.9)
Retinopathy of prematurity (n = 540)	57 (10.6)
Outcome	
Demised	15 (3.1)

## Discussion

Globally, prematurity and the associated low birth weight are one of the leading causes of neonatal morbidity and mortality [1,2].

Antenatal care plays a significant role in a neonate’s outcome and is associated with better survival to hospital discharge [14,15]. Antenatal care allows prevention, early identification and management of a multitude of risk factors, including HIV, syphilis and maternal hypertension, therefore decreasing the risk of prematurity and VLBWI mortality and morbidity. Thus, it is reassuring to see that 85% of mothers in this study did receive antenatal care at some stage during their pregnancy.

Maternal risk factors for preterm labour and low birth weight

Maternal hypertension, a partially modifiable risk factor, accounts for almost 18% of maternal deaths in South Africa, with 75% of those deaths thought to be preventable [11]. It has also been shown to increase the risk of low birth weight and prematurity, with some reported incidences of low birth weight being as high as 24% [16]. This study found the frequency of gestational hypertension to be 33.7%, which is higher than the nationally reported rates of approximately 12.5% and global rates of 2%-8% [17,18]. The increased rate in this sample is most likely due to the fact that the study was done at a tertiary level facility, which mostly caters to complicated cases.

Maternal HIV infection has been shown to not have any adverse effects on the outcomes of neonates once delivered [19]; however, it has been shown to put mothers at higher risk for preterm labour and low-birth-weight infants [20]. The rate of HIV infection found in this study was 26.4%, making it an important risk factor in our population. Further investigation is required to assess whether a correlation between the stage of disease and the risk of these adverse outcomes exists. Prevention of early mother-child HIV transmission should be a priority in LMICs.

Africa has the highest prevalence of maternal syphilis, accounting for 63.1% of the total infections, with a prevalence of 1.68% [21]. Syphilis has been shown to increase the risk of preterm labour and prematurity, along with other adverse effects such as stillbirth and congenital syphilis infections [21]. In our study, the high prevalence of maternal syphilis (2.9%) was very concerning. This could be related to the delayed presentation of some mothers in labour or the global shortage of penicillin antibiotics and the availability of treatment at local clinics [22].

Benefits of KMC

The KMC unit admitted VLBWIs greater than 1200 g. This study highlights that KMC should not have a weight cutoff, provided that the neonate is clinically and haemodynamically stable. However, the higher birth weight and gestational age admission criteria to the KMC unit could be associated with improved survival and better outcomes compared to studies done in the same unit [15].

In this study, the average rate of weight gain (37.6 g/kg/day) surpasses the recommended rate for the third trimester of intrauterine life (15-20 g/kg/day) [6], as well as the reported rates of conventional care VLBW neonates (i.e., non-KMC patients), which is between 13 and 18 g/kg/day [23,24]. For comparison, rates in other KMC samples range from 10 to 24 g/kg/day [25]. The majority of VLBWIs were breastfed (61.5%), which was most likely attributed to optimal weight gain. However, the breastfeeding rates could be vastly improved.

Two meta-analyses comparing outcomes of KMC and conventional care infants have shown no statistically significant difference in the rate of weight gain between the two samples; however, some studies do report at least small differences between the two [5,26,27]. Ideally, a second control cohort would need to be included in this study to accurately compare the rates.

The mean duration of stay was 37 days, which was shorter than described in another South African facility and other low- and middle-income countries (LMICs), which were between 39 and 45 days [28,29]. High-income countries (HICs) report longer durations, up to 63 days [29]. Data regarding the impact of KMC on the length of hospital stay has shown that while there is generally a decrease in the duration, that it is not always statistically significant [5,30]. Interestingly, a meta-analysis done in 2016 found that early implementation of KMC did have a significant impact on the duration of stay [26]; however, this is not always feasible, depending on the clinical situation and resource limitation. The duration of admission of these infants is directly affected by their comorbidities and the rate of weight gain, which is likely to be faster in KMC.

Complications of prematurity

The overall mortality for this sample was low ( 3.1%). This is significantly lower than those VLBWIs in the neonatal unit in the same hospital [14]. No difference in mortality was found between early implementation (i.e., immediately) and implementation only once stable [5].

Respiratory distress syndrome on admission is a common and expected complication of prematurity. The prevalence in this cohort was almost 84%, mirroring studies done in similar socio-economic settings on similar cohorts [13]. The high rates of surfactant (63.8%) and nasal continuous positive airway pressure (NCPAP) (67.3%) administration are in keeping with this, and the introduction of surfactant therapy has led to a drastic decrease in mortality in HICs [31]. Despite these interventions, the overall mortality from respiratory-related diseases in neonates can be as high as 60% in LMICs, approximately 10-fold higher than those reported in HICs [32]. However, the admission criteria for the unit, and therefore inclusion criteria for this study, includes stability on room air, therefore negating the severe forms of the disease and explaining the significantly lower mortality in our sample.

Late-onset sepsis was noted in 26.1% of the patients admitted to the unit. The rates of sepsis in other studies range from 12.2% up to 24% (culture-confirmed) but can go as high as 52% if the clinical diagnosis is included [33-35]. KMC has been shown to lower the risk of sepsis significantly with a meta-analysis done by Boundy et al. suggesting by as much as 47% [5]. Sepsis is both a major contributor to mortality in neonates (as high as 35.5% in LMICs), as well as a contributor to a prolonged duration of hospital stay [15]. The high rate of sepsis noted in this sample is thus concerning, and careful attention needs to be paid to infection prevention and control.

Retinopathy of prematurity (ROP) is a vaso-proliferative disorder of the retina, with a multifactorial risk factor profile, including oxygen therapy [36,37]. The prevalence of ROP was low (10.6%); this could be attributed to the fact that the cohort of babies did not require a prolonged duration of oxygen therapy. Similarly, a study found that those between one and five weeks of age were at increased risk of ROP if receiving at least two weeks of oxygen to attain saturation between 91% and 96%, while those between six and nine weeks old were at risk if they spent three weeks on oxygen for saturation above 97% [37].

Limitation

This was a single-centre retrospective study. It was difficult to retrieve some missing information from the electronic database because patient files were archived six weeks after discharge. The weight criteria for admission to KMC is greater than 1200 g, which could result in improved survival and decreased morbidity.

## Conclusions

Kangaroo Mother Care is a versatile and simple intervention capable of filling the gap in a wide variety of socio-economic settings. The benefits of its implementation are comprehensive, including low mortality, a greater rate of weight gain, decreased duration of hospital admission and lower prevalence of a number of complications of prematurity such as late-onset sepsis. The expansion of KMC programs locally and abroad is justified, however, more detailed research locally comparing KMC with conventional care programs is warranted.
